# Supplementary material for: Preinitiation Complex Loading onto mRNAs with Long versus Short 5′ TLs
Source: Int J Mol Sci. 2022 Nov 2;23(21):13369. doi: 10.3390/ijms232113369 (PMC9658832; doi:10.3390/ijms232113369)
Supplement: Supplementary file 1 [file ijms-23-13369-s001.zip › ijms-1957850-supplementary.pdf]

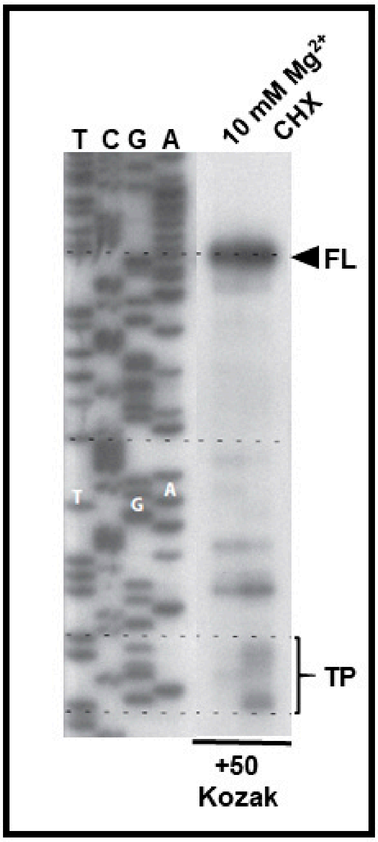

Figure S1: Toe-printing on a long 5' TL with a Kozak context.
The 80S toe-print (TP) was prepared in the presence of CHX. The position of the AUG start codon and the full-length (FL) reverse transcription product are indicated. As a control, the RRLs were supplemented with 10 mM MgOAc that inhibits ribosome loading.

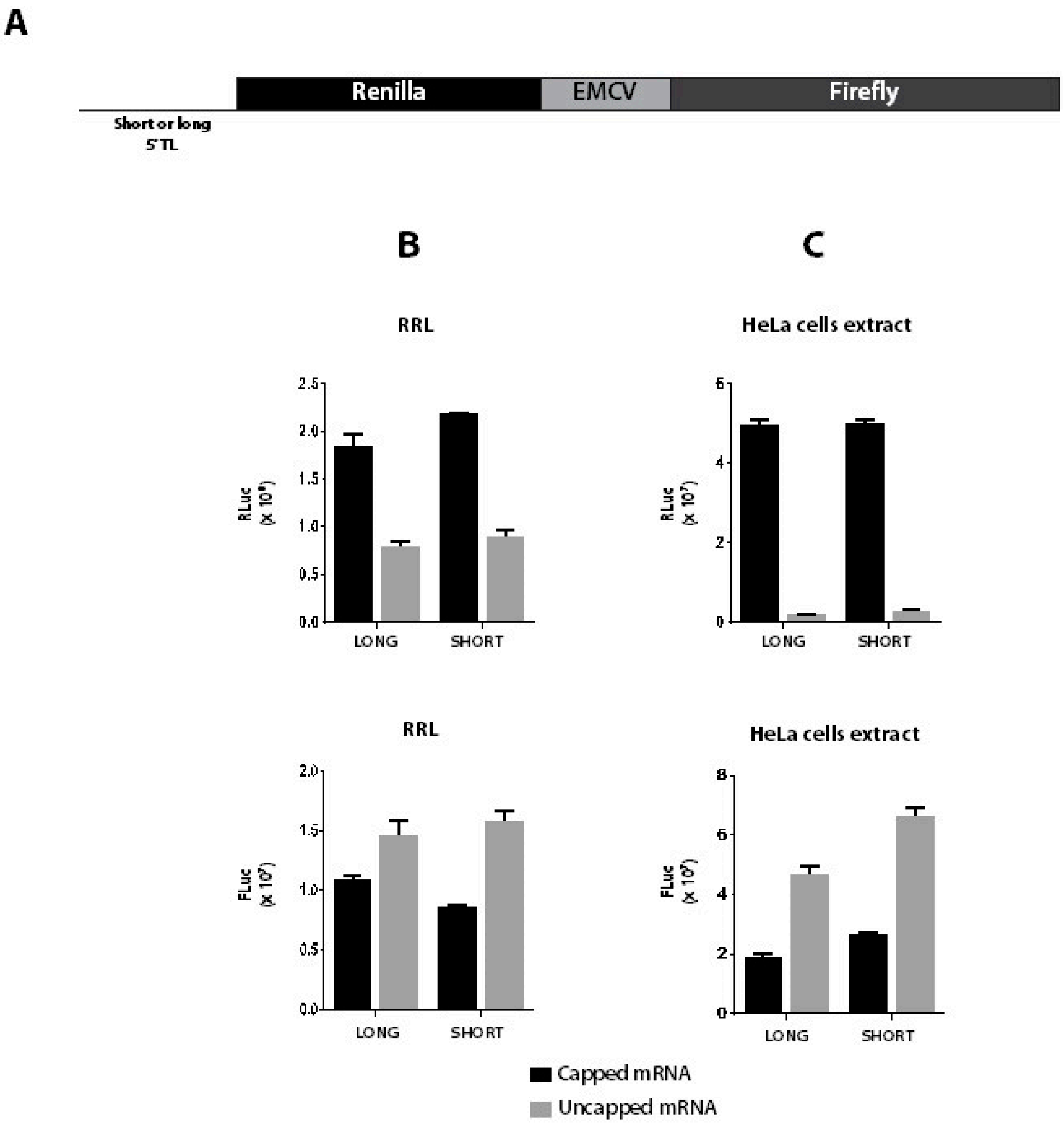

Figure S2: Translation from a short 5' TL is cap-dependent.

A. Schematic representation of the bicistronic mRNA with Renilla under cap-dependent translation and Firefly under the EMCV IRES.

B. Renilla and Firefly luciferases assay. Cap or uncapped messenger RNAs were incubated in rabbit reticulocyte lysate (RRL) or HeLa cell extract. Reporter activities are indicated in arbitrary units. Bars represent the SEM from triplicates.
